# Factors associated with suicidal ideation in the French nationwide EPICOV study

**DOI:** 10.1192/j.eurpsy.2022.1365

**Published:** 2022-09-01

**Authors:** C. Davisse-Paturet, M. Orri, A.-M. Florence, J.-B. Hazo, M.-C. Geoffroy, M. Melchior, A. Rouquette

**Affiliations:** 1 Inserm U1018 - CESP, Equipe Psychiatrie Du Développement Et Trajectoire, Paris, France; 2 McGill University, Department Of Psychiatry, Rue Sherbrooke, Canada; 3 INSERM, Sorbonne University, Team Of Social Epidemiology (eres), Pierre Louis Institute Of Epidemiology And Public Health (iplesp), Paris, France; 4 Ministère des Solidarités et de la Santé, Drees, Paris, France

**Keywords:** suicidal ideation, France, COVID19

## Abstract

**Introduction:**

The COVID-19 pandemic seems associated with a worsening in mental health issues as well as a widening of pre-existing social and health inequalities. Assessment of its impact on suicidal behavior might therefore be relevant.

**Objectives:**

To assess factors associated with suicidal ideation in the general population, using data from the French nationwide Epicov study

**Methods:**

In the nationally representative Epicov study, data on occurrence of suicidal ideation from November 2019 to November 2020 were available, including timing with respect to France’s lockdown periods. We studied the incidence of suicidal ideation among participants without a prior history of suicidal behavior, after May 11, 2020, when first COVID-19 related lockdown was suspended. Studied factors assessed sociodemographic and health status, including mental health, as well as COVID-19 related information such as symptom. Associations between selected factors and suicidal ideation were assessed in sex-stratified adjusted logistic regression models.

**Results:**

In the Epicov study, prevalence of suicidal ideation was of 2,9%, as opposed to around 4% pre-pandemic. Among 48 702 female and 41 016 male participants, health or financial issues were associated with a higher incidence of suicidal ideation. Interestingly, Covid19-like symptoms were also associated with higher risk of suicidal ideation. While an impaired mental health has been observed in survivors of past pandemics, given the unprecedented context of the current pandemic, this association needs further investigation.

**Conclusions:**

While the COVID-19 pandemic might have lowered suicidal ideation, it’s aftermaths may reverse the trend. To prevent this rise, identification of vulnerable groups is crucial to promote tailored public health strategies.

**Disclosure:**

No significant relationships.

